# The Association of Individual and Neighborhood Social Cohesion, Stressors, and Crime on Smoking Status Among African-American Women in Southeastern US Subsidized Housing Neighborhoods

**DOI:** 10.1007/s11524-014-9911-6

**Published:** 2014-10-15

**Authors:** Jeannette O. Andrews, Martina Mueller, Susan D. Newman, Gayenell Magwood, Jasjit S. Ahluwalia, Kellee White, Martha S. Tingen

**Affiliations:** University of South Carolina, Columbia, SC USA

**Keywords:** African-American women, Subsidized housing, Smoking, Social context, Health disparities

## Abstract

The purpose of this study was to examine the associations between individual and neighborhood social contextual factors and smoking prevalence among African-American women in subsidized neighborhoods. We randomly sampled 663 adult women in 17 subsidized neighborhoods in two Southeastern US states. The smoking prevalence among participants was 37.6 %, with an estimated neighborhood household prevalence ranging from 30 to 68 %. Smokers were more likely to be older, have lower incomes, have lower BMI, and live with other smokers. Women with high social cohesion were less likely to smoke, although living in neighborhoods with higher social cohesion was not associated with smoking prevalence. Women with higher social cohesion were more likely to be older and had lived in the neighborhood longer. Women with high stress (related to violence and disorder) and who lived in neighborhoods with higher stress were more likely to smoke. Younger women were more likely to have higher stress than older women. There were no statistically significant associations with objective neighborhood crime data in any model. This is the first study to examine both individual and neighborhood social contextual correlates among African-American women in subsidized neighborhoods. This study extends findings about smoking behaviors and neighborhood social contexts in this high-risk, urban population. Future research is needed to explore age and residential stability differences and perceptions of social cohesion, neighborhood disorder, and perceived violence in subsidized housing. Further research is also warranted on African-American women, subsidized housing, smoking, social context, health disparities’ effective strategies to address these individual and contextual factors to better inform future ecological-based multilevel prevention, and cessation intervention strategies.

Ethnic minority women have high rates of tobacco-related diseases and resultant health disparities.^1^,^2^ Understanding and reducing this burden remains a national priority.^3^,^4^ The smoking prevalence among urban African-American women living in subsidized housing neighborhoods has been reported as high as 60 %,^5^^–^ 7 which is three times higher than African-American women who smoke in the general population (19 %).^8^ To better understand these wide variances among subgroups, there is an increasing focus to examine both individual and neighborhood social contextual influences on behaviors.^9^^–^ 14 Social ecological perspectives of health posit that individual, social, and neighborhood factors converge to influence health behaviors, such as smoking.^15^

There are currently 1.2 million households living in US public housing and 3.1 million households receiving Section 8 vouchers.^16^ The majority of these households are led by single women.^16^ Residents of subsidized housing neighborhoods are among the most impoverished in the USA and often experience high stress, neighborhood disorder, high violence, and poor physical and mental health.^17^^–^ 19 In poor urban neighborhoods, women face stressors of densely populated households, concentrations of unemployment, and low education, all of which are established social determinants associated with smoking.^11^,^20^ Living in deprived and disordered neighborhoods can create a sense of danger and uncertainty and heighten other social conflicts with family, peers, and neighbors.^21^ Neighborhood disorder may be a source of chronic stress that contributes to unhealthy behaviors such as smoking.^22^,^23^

Urban subsidized housing neighborhoods historically have experienced high occurrences of crime and violence.^24^ Women in subsidized housing have higher victimization rates and higher fear of crime than women in higher social classes.^24^ Self-reported neighborhood violence among African-American pregnant women has been linked with early pregnancy cigarette use.^25^ There are no studies that report the association with stressors of perceived neighborhood disorder and violence, and neighborhood crime data with smoking among African-American women in subsidized housing.

Conversely, social environmental factors such as neighborhood cohesion have been associated with lower levels of smoking in whites^26^ and Asian-American neighborhoods,^12^ yet this has not been studied among African-American women in subsidized housing. Social cohesion, a component of social capital that reflects the connectedness of the community, may serve as a protective function related to health behaviors, both at the individual and the neighborhood level.^26^

Research is needed to understand both individual and neighborhood level stressors and potential protective factors among low-income minority urban women to inform ecologically based prevention and cessation interventions. To address these gaps, we sought to examine associations between social cohesion, stress from violence and neighborhood disorder, crime and smoking prevalence among African-American women living in subsidized neighborhoods on both, the individual and the neighborhood level. We hypothesized the following:

1. Women experiencing higher social cohesion and lower stress from violence and disorder would be less likely to smoke than women with low social cohesion and high stress.
2. Women living in neighborhoods with higher social cohesion, lower neighborhood stress from violence and disorder, and lower neighborhood crime would be less likely to smoke than their counterparts in subsidized neighborhoods with low social cohesion, high stress, and high crime.

## Methods

### Study Design and Sample

This study analyzed baseline data from a larger randomized controlled trial that tested effects of a multilevel cessation intervention in subsidized housing neighborhoods.^7^ Data reported here reflect baseline data prior to the conduct of the intervention. Seventeen urban subsidized neighborhoods in Charleston, SC, and Augusta, GA, were the primary sampling units and individuals within housing sites served as the secondary sampling units.

In 2009, Charleston and Augusta had 34 public housing and Section 8 neighborhoods. Neighborhood inclusion criteria were public housing or Section 8 neighborhoods, family residential neighborhoods (vs senior only), and at least 60 units/homes in a clustered site. Seventeen of the 34 neighborhoods met eligibility, and all 17 agreed to participate.

There were a total of 3,252 housing units in the 17 neighborhoods. We collected individual data from 20 % of randomly selected households in each neighborhood. Individual inclusion criteria included female head of household and over 18 years of age. We obtained a diagram of the layout of each housing neighborhood with unit household numbers. From this, the study biostatistician generated a uniform random number using SAS 9.2^27^ and assigned a number to each household. Households were sorted in ascending order by their corresponding random number. From this list, research assistants approached female heads-of-households (up to three visits on different days and times) for participation in order of randomization until the number of households required in each neighborhood had been surveyed. After providing an information sheet and obtaining verbal consent, the research assistant read aloud the surveys and marked participant’s responses on the data collection tool while at the respective household. Each woman completing the survey received a $10 gift card. The Medical University of South Carolina and Georgia Regents University institutional review boards approved all study procedures.

### Study Measures

#### Dependent Variable

The main dependent variable was self-reported current smoking. Individuals were classified as current smokers if they answered yes to both of the following interview questions: Have you smoked at least 100 cigarettes in your lifetime? Do you currently smoke cigarettes?

Individuals were considered nonsmokers if they answered no to the latter question. For additional descriptive purposes, we classified former smokers if they answered yes to question 1 and no to question 2, and we classified never smokers if answered no to both questions.

#### Independent Variables

##### Sociodemographics

Demographic variables included age; race/ethnicity; education level, employment, marital status, years living in neighborhood; current health, health care coverage, and annual household income.

##### Body Mass Index

Body mass index (BMI) was calculated as weight in kilograms divided by height in meters squared (weight (kg) / [height (m)]^2^). Height and weight were self reported.

##### Neighborhood Cohesion

Individual scores for neighborhood cohesion were evaluated using the 12-item Sense of Community Scale (SCS).^28^,^29^ This low-literacy scale has previously been validated with urban samples^28^,^29^ and allows for the neighborhood as the reference point for cohesion. This self-report survey consists of 12 true/false items that measure four dimensions: (1) membership (i.e., I can recognize most people who live in my neighborhood, I feel at home in this neighborhood), (2) influence (i.e., I care about what my neighbors think of my actions, if there is a problem in this neighborhood people who live here can get it solved), (3) reinforcement of needs (i.e., I think my neighborhood is a good place for me to live, my neighbors and I want the same things from the neighborhood), and (4) shared emotional connection (i.e., it is very important for me to live in this particular neighborhood, I expect to live in this neighborhood for a long time). Total scores reflect a sum of the items and range from 0 to 12 and subscales scores ranged from 0 to 3, with higher scores indicating higher cohesion. Reported internal consistency ranges from 0.72 to 0.80 and acceptable construct validity.^28^,^29^ Cronbach’s alpha in this study was 0.74.

To estimate neighborhood level cohesion scores, a mean of the total scores was calculated for each neighborhood. Tertiles (i.e., low, medium, high) were created based on total scores using the entire sample distribution.

##### Neighborhood Stress

Individual scores for neighborhood stress were measured with the City Stress Index (CSI), an 18-item, four-point Likert scale.^21^ This low literacy stress scale has been tested with low-income urban groups to measure contextual neighborhood characteristics that make life stressful, particularly perceived neighborhood disorder and exposure to violence.^21^ Self-report was used to measure the two subscales: (1) neighborhood disorder (i.e., observing people selling drugs, adults arguing on the street, knowing someone in jail, harassment by police) and (2) exposure to violence (i.e., family member attacked, family member stabbed or shot, family member robbed). Responses are never, once, few times, and often from stress experienced in the neighborhood in the past year. Total scores are summed and range from 18 to 72, neighborhood disorder subscale ranges from 11 to 44, and exposure to violence subscale ranges from 7 to 28, with higher scores indicating higher stress. Reported internal consistency were Cronbach’s alpha 0.88 for neighborhood disorder and 0.85 for exposure to violence. The 1-year test-retest correlation coefficients (Pearson’s *r*) were 0.82 and 0.75. Construct validity has been based on factor analyses.^21^ Cronbach’s alpha in this study was 0.86 for the total scale and ranged from 0.82 for the neighborhood disorder subscale to 0.83 for the violence subscale.

To estimate neighborhood level stress scores, a mean of the total and subscale scores was calculated for each neighborhood. Tertiles (i.e., low, medium, high) were created based on total scores using the entire sample distribution.

##### Neighborhood Crime Data

Actual crime data for each neighborhood were collected from the corresponding police departments for a 1-year period prior to survey data collection in each neighborhood. Similar to McDonald,^30^ we grouped crime into three categories: property (arson, burglary, theft, and vandalism), violent (murder, robbery, assault, and battery), and quality of life (weapon offenses, prostitution, drug arrest, liquor law violations, and disorderly conduct). Due to the variance in size of the neighborhoods (61 units to 450 units), we calculated a crime rate proportion by dividing the actual number of crimes for each category and total by the number of units in the neighborhood. Tertiles (i.e., low, medium, high) were created based on total crime proportions using the entire sample distribution.

##### Neighborhood Household Smoking Prevalence

The neighborhood household smoking prevalence was calculated for each neighborhood based on the proportion of individuals who self-reported smoking or reported a member of the household smoking from the total sample surveyed in each neighborhood.

### Analyses

The sample was described using simple summary statistics (means for continuous variables and proportions for categorical variables). For descriptive analysis, the sample was divided into three groups based on smoking status (i.e., current smoker, former smoker, and never smoker). Since the focus of our study was current smoking, we compared groups (current smokers vs nonsmokers) with respect to demographic, clinical, and neighborhood characteristics using independent sample *t* tests for continuous variables and chi-squared tests for categorical variables.

For investigation of the relationship of selected covariates with smoking status, two groups were compared: current smoker versus nonsmoker. Bivariate models were used with smoking status as dependent variable and demographic, clinical, and neighborhood characteristics individually as independent variables. Subsequently, two sets of generalized linear mixed models (generalized estimation equation (GEE)-type models) were developed to evaluate the relationship between smoking status and individual, as well as neighborhood level variables (fixed effects). The first set consisted of only individual level variables while the second set included both individual and neighborhood level variables. To take clustering of participants within the 17 neighborhoods into account, models were adjusted for study neighborhood (i.e., the models included neighborhood as random effect). Neighborhood level variables included the mean total crime proportion, mean total scores within neighborhoods on the stress and cohesion scales, and neighborhood smoking prevalence. Stress and cohesion scales were first assessed for normality; scores were transformed as appropriate or quadratic centered terms were included if nonlinearity was determined. Models were developed initially using neighborhood cohesion, stress, and crime proportion individually while adjusting for demographic and clinical variables due to high correlation between the stress and cohesion scores; only the full models in both sets are presented.

In additional analyses, neighborhood stress, cohesion scores, and crime proportions were categorized into low, moderate, and high using tertiles of the mean total scores. Smoking status, mean age, and mean number of years lived in the neighborhood were compared across these three variables using independent sample *t* tests for continuous variables (dichotomized into high vs low) and chi-squared tests for categorical variables.

All significance tests were two-sided with a significance level of α < 0.05. Analyses were conducted using SAS version 9.2.^27^

## Results

### Participant Characteristics

Approximately 800 households in the 17 subsidized housing neighborhoods were approached for data collection from the randomized list of households in each neighborhood. Of these, less than 2 % (*n* = 15) were excluded due to no woman living in the home, and approximately 72 households (9 %) were excluded due to no answer at the door after three attempts over a 2-week period on varying days/times of day approached (i.e., morning, early afternoon, late afternoon). Less than 5 % (40 women) who were approached refused to participate in the survey.

A total of 663 adult women participated in the survey; of those, 507 women provided information to all questions and had no missing data. The majority in the sample was African-American (92 %), with an average age of 39 years. Most women were single or never married (67 %) and 85 % had a high school education or less. Less than one third (31 %) reported working full- or part-time, and 58 % reported a total household income of less than $10,000 annually. On average, women lived in their respective neighborhood for 5 years. More than one of every three women, or 37.6 %, self-reported smoking.

### Individual Level Correlates of Smoking Behaviors

Demographic variables associated with current smoking included age, income, BMI, and smokers in the household as described in Table 1. Race was not analyzed since the majority of participants were African-American. Current smokers were more likely to be older (*χ* ^2^ = 13.6, *p* = .009), have lower incomes (*χ* ^2^ = 13.7, *p* < 0.001), and lower BMI [*M* = 29.6 (SD = 8.0)] as compared to nonsmokers [31.9 (SD = 9.2); *t* = 3.3, *p* = .008]. Current smokers were more likely to have other smokers in the household than nonsmokers [*M* = 1.3 (SD = 0.6) vs 0.2 (SD = 0.6); *t* = −22.8, *p* < 0.001)].

**TABLE 1:** Association between study variables and smoking status (*N* = 663)

	Current smoker (*N* = 249)	Nonsmoker (*N* = 414)		*p* value^a^
		Former smoker (*N* = 57)	Never smoker (*N* = 357)	
Age	*M* = 37.8 (14.2)	*M* = 50.6 (17.1)	*M* = 37.6 (17.3)	0.188^b^
Age				0.009^c^
18–29 (*n* = 260)	*N* = 89 (35.7 %)	*N* = 11 (19.3 %)	*N* = 160 (44.8 %)	
30–39 (*n* = 135)	*N* = 59 (23.7 %)	*N* = 7 (12.3 %)	*N* = 69 (19.3 %)	
40–49 (*n* = 89)	*N* = 41 (16.5 %)	*N* = 6 (10.5 %)	*N* = 42 (11.8 %)	
50–64 (*n* = 124)	*N* = 49 (19.7 %)	*N* = 21 (36.8 %)	*N* = 54 (15.1 %)	
>65 (*n* = 55)	*N* = 11 (4.4 %)	*N* = 12 (21.1 %)	*N* = 32 (9.0 %)	
Marital status				0.539^c^
Single/never married (*n* = 447)	*N* = 178 (73.0 %)	*N* = 24 (43.6 %)	*N* = 245 (71.2 %)	
Member of unmarried couple (*n* = 3)	*N* = 1 (0.4 %)	*N* = 0 (0 %)	*N* = 2 (0.6 %)	
Married (*n* = 35)	*N* = 11 (4.5 %)	*N* = 2 (3.6 %)	*N* = 22 (6.4 %)	
Separated (*n* = 44)	*N* = 17 (7.0 %)	*N* = 7 (12.7 %)	*N* = 20 (5.8 %)	
Divorced (*n* = 65)	*N* = 24 (9.8 %)	*N* = 14 (25.5 %)	*N* = 27 (7.9 %)	
Widowed (*n* = 49)	*N* = 13 (5.3 %)	*N* = 8 (14.6 %)	*N* = 28 (8.1 %)	
Educational degree				0.212^c^
Less than high school (*n* = 199)	*N* = 77 (30.9 %)	*N* = 23 (40.4 %)	*N* = 99 (27.7 %)	
High school diploma or equivalent (*n* = 366)	*N* = 135 (54.2 %)	*N* = 24 (42.1 %)	*N* = 207 (60.0 %)	
Associate’s degree (*n* = 36)	*N* = 10 (4.0 %)	*N* = 5 (8.8 %)	*N* = 21 (5.9 %)	
Bachelor’s degree (*n* = 16)	*N* = 5 (2.0 %)	*N* = 1 (1.8 %)	*N* = 10 (2.8 %)	
Master’s degree (*n* = 3)	*N* = 0	*N* = 0	*N* = 3 (0.8 %)	
Other (*n* = 43)	*N* = 22 (8.8 %)	*N* = 4 (7.0 %)	*N* = 17 (4.8 %)	
Highest grade in school	M = 11.6 (1.7)	*M* = 10.9 (2.4)	*M* = 11.8 (1.9)	0.430^b^
Working status				0.057^c^
Working full time (*n* = 137)	*N* = 45 (18.1 %)	*N* = 8 (14.0 %)	*N* = 84 (23.5 %)	
Working part time (*n* = 70)	*N* = 28 (11.2 %)	*N* = 5 (8.8 %)	*N* = 37 (10.4 %)	
Unemployed/laid off (*n* = 110)	*N* = 47 (18.9 %)	*N* = 7 (12.3 %)	*N* = 56 (15.7 %)	
Looking for work/unemployed (*n* = 121)	*N* = 55 (22.1 %)	*N* = 6 (10.5 %)	*N* = 60 (16.8 %)	
Student (*n* = 68)	*N* = 24 (9.6 %)	*N* = 4 (7.0 %)	*N* = 40 (11.2 %)	
Disabled (*n* = 116)	*N* = 43 (17.3 %)	*N* = 21 (36.8 %)	*N* = 52 (14.6 %)	
Retired (*n* = 33)	*N* = 5 (2.0 %)	*N* = 5 (8.8 %)	*N* = 23 (6.4 %)	
Other (*n* = 8)	*N* = 2 (0.8 %)	*N* = 1 (1.8 %)	*N* = 5 (1.4 %)	
Income				0.008^c^
$0–$5,000 (*n* = 275)	*N* = 117 (62.6 %)	*N* = 20 (45.5 %)	*N* = 138 (49.1 %)	
$5,001–$10,000 (*n* = 107)	*N* = 35 (18.7 %)	*N* = 11 (25.0 %)	*N* = 61 (21.7 %)	
$10,001–$20,000 (*n* = 85)	*N* = 22 (11.8 %)	*N* = 9 (20.5 %)	*N* = 54 (19.2 %)	
$20,001–$30,000 (*n* = 28)	*N* = 11 (5.9 %)	*N* = 2 (4.5 %)	*N* = 15 (5.3 %)	
$30,001–$40,000 (*n* = 17)	*N* = 2 (1.1 %)	*N* = 2 (4.5 %)	*N* = 13 (4.6 %)	
BMI	*M* = 29.6 (8.0)	*M* = 33.5 (12.1)	*M* = 31.6 (8.7)	0.001^b^
Current health				0.264^c^
Excellent (*n* = 124)	*N* = 39 (16.0 %)	*N* = 10 (18.2 %)	*N* = 75 (21.8 %)	
Good (*n* = 293)	*N* = 110 (45.1 %)	*N* = 19 (34.5 %)	*N* = 164 (47.7 %)	
Fair (*n* = 188)	*N* = 80 (32.8 %)	*N* = 18 (32.7 %)	*N* = 901 (26.2 %)	
Poor (*n* = 38)	*N* = 15 (6.2 %)	*N* = 8 (14.5 %)	*N* = 15 (4.4 %)	
Health care coverage (yes: *n* = 461)	*N* = 164 (67.5 %)	*N* = 46 (83.6 %)	*N* = 251 (67.5 %)	0.051^c^
Years lived in neighborhood	*M* = 4.7 (6.7)	*M* = 5.6 (5.8)	*M* = 5.5 (8.4)	0.144^b^
Number smokers in household	*M* = 1.3 (0.6)	*M* = 0.4 (0.8)	*M* = 0.2 (0.5)	<0.0001^b^
Section 8 (yes: *N* = 158)	*N* = 59 (37.3 %)	*N* = 14 (24.6 %)	*N* = 85 (23.8 %)	0.949^c^
Sense of Community Scale (SCS) total score	*M* = 6.2 (3.0)	*M* = 6.9 (3.1)	*M* = 6.3 (2.8)	0.272^b^
City Stress Inventory (CSI) total score	*M* = 36.8 (9.6)	*M* = 33.0 (9.3)	*M* = 33.0 (10.0)	<0.0001^b^

^a^ *p* values are reported for smoker versus nonsmoker

^b^From *t* test

^c^From chi-squared test (columns may not add to 100 % due to rounding error)

### Neighborhood Characteristics

Neighborhoods ranged in size from 61 units to 356 units as shown in Table 2. The mean neighborhood household smoking prevalence was 47.7 %, with ranges from 30 to 68.5 %. Neighborhood social cohesion ranged from 3.8 to 9.2, with a mean of 6.4 (SD = 4.1). Neighborhood stress (e.g., disorder and crime exposure) scores ranged from 25.7 to 44.8, with a mean score of 33.9 (*SD* = 4.1). Mean neighborhood actual crime proportions were 45.1 % (*SD* = 27) ranging from 10.8 to 115.5 %.

**TABLE 2:** Smoking prevalence, social cohesion, stress, and crimes by neighborhood

	*N* = total no. of homes	*n* = no. of surveyed	Neighborhood smoking prevalence (no. of smoking households/*n*)	Mean social cohesion scale (SCS)	Mean city stress index (CSI)	Total no. of crimes/annual	Crime proportion (no. of crimes/no. of homes)	Violent crime proportion (no. of violent crimes/no. of homes)
1	272	54	68.5 %	4.5	44.8	69	0.25	0.05
2	257	53	45.3 %	7.2	32.7	43	0.17	0.02
3	178	35	60.0 %	6.1	36.2	27	0.15	0.06
4	201	40	67.5 %	5.3	38.4	113	0.56	0.09
5	61	20	30.0 %	7.7	31.6	13	0.21	0.03
6	356	70	45.7 %	6.9	33.7	52	0.15	0.03
7	74	20	35.0 %	9.2	29.6	49	0.66	0.14
8	254	50	40.0 %	6.9	32.6	91	0.36	0.16
9	100	20	55.0 %	5.9	35.1	73	0.73	0.07
10	246	50	52.0 %	6.5	33.3	166	0.68	0.14
11	149	30	36.7 %	6.3	31.4	76	0.51	0.11
12	142	27	37.0 %	6.5	30.3	164	1.15	0.30
13	352	70	41.4 %	5.6	35.1	109	0.31	0.04
14	144	31	48.4 %	6.4	34.8	82	0.57	0.17
15	150	30	50.0 %	5.7	35.0	293	0.65	0.13
16	166	33	30.3 %	9.0	25.7	18	0.11	0.02
17	150	30	50.0 %	3.8	36.6	68	0.45	0.13
Total/mean (SD)	3,252	663	47.7 % (SD = 10.9)	6.4 (SD = 4.1)	33.9 (SD = 4.1)	88.6 (SD = 68.5)	0.45 (SD = 0.27)	0.10 (SD = 0.07)

### Neighborhood Level Correlates with Smoking Behavior

Social cohesion and neighborhood stress total scores were highly correlated (*r* = −0.45, *p* < 0.0001) for individual level scores and for mean scores across neighborhoods (*r* = −0.87, *p* < 0.0001). No statistically significant correlation was observed between actual crime proportions and self-reported neighborhood stress (disorder and violence exposure) or cohesion scores. Therefore, neighborhood level variables were examined individually adjusted for demographic and clinical covariates.

Social cohesion total scores and three of the subscales (e.g., influence, needs, and connection) were statistically significantly associated with current smoking in logistic models as shown in Table 3, with those with higher social cohesion less likely to smoke. As shown in Table 4, women who perceived living in neighborhoods with high social cohesion (vs low to moderately cohesive neighborhoods) were likely to be older (mean age = 46.4 years, SD = 19) and lived in the neighborhood longer (mean = 7.5 years, SD = 10.1). However, no statistically significant differences in smoking prevalence were observed based on living in low, moderate, or high socially cohesive neighborhoods as shown in Table 4.

**TABLE 3:** Bivariate generalized linear mixed models with neighborhood correlates and odds of smoking (*N* = 663)

	Odds ratio	95 % CI	*p* value
Sense of Community Scale (SCS) total score	0.75	0.63; 0.90	0.0023
Membership subscale	0.92	0.44; 1.91	0.8120
Influence subscale	0.26	0.13; 0.54	0.0003
Needs subscale	0.32	0.16; 0.61	0.0007
Connection subscale	0.44	0.26; 0.72	0.0013
City stress index (CSI) total score:	1.01	1.00; 1.02	<0.0001
Violence subscale^a^			
Linear term	1.07	1.02; 1.12	0.0030
Quadratic term	1.00	1.00; 1.00	0.0096
Disorder subscale	1.01	1.01; 1.02	<0.0001
Total crime proportions	0.99	0.99; 1.01	0.6500
Violent crime proportions	0.98	0.95; 1.01	0.1780

^a^The odds of being a current smoker increased by 7 % for every one unit increase in violence subscale score, but this increase is decelerated by about 0.2 % for each one unit increase in score

**TABLE 4:** Comparison of smoking status, age and time in neighborhood across tertiles for sense of community, city stress index, and neighborhood crime (*N* = 663)

Sense of Community Scale (SCS) total score (tertiles)				
	5.6 or less (lower)	5.7–7.4 (moderate)	7.5 or above (higher)	*p* value
Current smoker (yes)	39.4 % (99/251)	39.6 % (63/159)	34.4 % (87/253)	0.4161^a^
Age	32.7 ± 11.9	36.4 ± 16.1	46.4 ± 18.0	<0.0001^b^
Years lived in neighborhood	3.8 ± 4.9	3.9 ± 4.9	7.5 ± 10.1	<0.0001^b^
City stress index (SCI) total score (tertiles)				
	32.1 or less (lower)	32.2–38.4 (moderate)	38.5 or above (higher)	
Current smoker (yes)	28.8 % (91/316)	40.3 % (52/129)	48.6 % (106/218)	<0.0001^a^
Age	43.0 ± 19.0	37.2 ± 14.1	33.7 ± 12.1	<0.0001^b^
Years lived in neighborhood	5.5 ± 8.3	4.7 ± 6.3	5.0 ± 7.1	0.5268^b^
Neighborhood crime level (tertiles)				
	Low	Moderate	High	
Current smoker (yes)	37.9 % (146/385)	37.2 % (86/231)	36.17 % (17/47)	0.9651^a^
Age	38.5 ± 17.4	39.4 ± 15.4	38.4 ± 15.3	0.8017^b^
Years lived in neighborhood	4.3 ± 5.6	7.1 ± 10.2	3.1 ± 3.2	<0.0001 ^b^

^a^High versus low; high versus moderate

^b^All comparisons

Neighborhood stress (total score) and both subscales (disorder and crime exposure) were statistically significantly associated with smoking in generalized linear mixed models as shown in Table 3. As shown in Table 4, women who reported living in high stress neighborhoods (vs low to moderate stress) were more likely to smoke (48.6 vs 28.8, 40.3 %, *p* < 0.0001). Younger women (mean = 33.7 years, SD = 12.1) were more likely to report their neighborhoods were high stress, compared to women in low stress neighborhoods (mean age = 43 years, SD = 19) and moderate stress (mean age = 37.2 years, SD = 14.1).

No statistically significant association of neighborhood crime with smoking was observed (Table 3). As shown in Table 4, neighborhoods with high crime rates demonstrated the highest turnover in residency, with women living there on average 3.1 years, compared to those with moderate crime rates (7.1 years) and low crime rates (4.3 years). Similarly, when examining the relationship of low, moderate, and high neighborhood crime rates with low, moderate, and high stress and/or cohesive neighborhoods, no significant differences were observed among the groups in these analyses.

### Association Between Smoking, Individual Factors, and Neighborhood Level Factors

The association between smoking, individual factors, and neighborhood factors are described in Table 5. Although high multicollinearity was observed between neighborhood covariates, results for the full model are similar to those obtained from models including only one of the three neighborhood variables adjusted for the same set of covariates as the full model. Therefore, results for the full models are reported.

**TABLE 5:** Random effects results for models 1 and 2 modeling odds to be smoker (*N* = 507)

	Model 1, OR, 95 % CI		Model 2, OR, 95 % CI	
Age (years)				
18–29 (Ref)	1.00		1.00	
30–39	1.14*	(1.02; 1.28)	1.15*	(1.03; 1.29)
40–49	1.06	(0.92; 1.22)	1.07	(0.931; 1.23)
50–64	1.10	(0.95; 1.27)	1.15	(0.965; 1.29)
≥65	0.89	(0.72; 1.11)	0.87	(0.718; 1.09)
Marital status				
Single/never married (Ref)	1.00		1.00	
Married/unmarried couple	0.97	(0.81; 1.15)	0.96	(0.80; 1.14)
Separated/divorced/widowed	1.02	(0.90; 1.14)	1.00	(0.90; 1.15)
Years of education	0.99	(0.97; 1.02)	1.00	(0.97; 1.02)
Work status (working vs not working)	1.01	(0.91; 1.11)	1.00	(0.91; 1.11)
Income (>$10,000 vs ≤$10,000)	0.88*	(0.79; 0.98)	0.90	(0.81; 1.00)
Health care (no vs yes)	1.02	(0.92; 1.12)	0.99	(0.90; 1.09)
Health status (excellent/good vs fair/poor)	0.93	(0.84; 1.02)	0.92	(0.83; 1.01)
Body mass index	0.99**	(0.98; 0.99)	0.99**	(0.98; 0.99)
Living situation (Section 8 vs public housing)	1.07	(0.95; 1.19)	1.12	(0.99; 1.26)
Years lived in neighborhood	0.99	(0.99; 1.00)	0.99	(0.99; 1.00)
Sense of community index (SCS)	1.01^a^	(0.99; 1.03)	1.00^b^	(0.92; 1.09)
City stress inventory (CSI)	1.01^a^***	(1.01; 1.03)	1.00^b^	(0.97; 1.04)
Neighborhood crime proportion	–	–	1.00	(0.99; 1.00)
Neighborhood smoking prevalence	–	–	1.01	(0.99; 1.02)

Model 1 only includes individual-level factors + random effect to account for clustering within neighborhood; model 2 includes individual- and neighborhood-level factors + random effect to account for clustering within neighborhood

*OR* odds ratio, *CI* confidence interval, *HS* high school

^a^Individual-level CSI and SCS total scores used for model 1

^b^Neighborhood-level mean total scores used for model 2

*P ≤ 0.05; **P ≤ 0.01; ***P ≤ 0.001

Table 5 reports results from the set of participants with complete data for all variables included in the generalized linear mixed models. In subsequent sensitivity analyses, results were compared to the full set of participants after imputation of missing data using multiple imputation methods. Results were similar for all data sets; therefore, the results for 507 participants with complete data are reported.

In the multivariate model 1, significant individual level factors associated with smoking were age, with 30–39 year-old women more likely to smoke [odds ratio (OR) = 1.14, 95 % confidence interval (CI) = 1.02–1.28, *p* ≤ .05] than younger women 18–29 years old. Women with a household annual income >$10,000 were less likely to smoke (OR = 0.88, 95 % CI = 0.79–0.98, *p* ≤ 0.05) than women with <$10,000 annual income. Women with higher body mass index were less likely to smoke (OR = .99, 95 % CI = 0.98–0.99, *p* ≤ 0.01), and women who perceived living in a high stress neighborhood were more likely to smoke (OR = 1.01, 95 % CI = 1.01–1.03, *p* ≤ 0.001). In other words, for a one-unit increase in BMI, women had 1 % lower odds of smoking while a one-unit increase in neighborhood stress resulted in a 1 % increase in odds to be a smoker holding all other variables in the model at fixed values.

When adjusting for neighborhood level factors, the results were similar, as shown in model 2 with age and BMI remaining significant as in model 1.

## Discussion

Tobacco use among African-American women living in subsidized neighborhoods is a major public health threat for these individuals, families, and communities. This is the first study to report neighborhood social contextual factors and smoking prevalence among African-American women in US subsidized housing. In our study, the individual level factors for smoking were similar to those in the Black Women’s Health Study, indicating that age (i.e., >30 years old) and low income are associated with current smoking in African-American women.^31^ Our findings support other findings that African-American women who smoked were more likely to live in a household with other smokers.^31^^–^ 33

These findings reveal high smoking prevalence among individual African-American women and these urban subsidized neighborhoods in the Southeastern USA. The contagion perspective is a construct that suggests that individuals are influenced by others in their environment, and behavior, such as smoking, may spread as a result of local norms, experiences, or information.^31^,^34^ Smoking behaviors are highly visible in dense neighborhoods with shared outdoor space, supporting a normative climate that diminishes the social stigma of otherwise unacceptable and undesirable health behaviors.^34^^–^ 36 National studies have consistently reported that African-American women in the Southeastern USA have the lowest smoking prevalence compared to African-American women in other regions.^30^,^37^,^38^ King and colleagues^38^ analyzed data from the National Health Survey over a decade ago and found that African-American women in the deep south (e.g., where this study took place) were significantly less likely to smoke than women in other regions in the USA. The findings in this study demonstrate the influence of neighborhood social contexts on smoking status among women African-American women living in urban, subsidized housing in the Southeastern USA.

Women with higher social cohesion were less likely to smoke in our study indicating a potential protective factor against smoking, although living in neighborhoods with higher social cohesion was not associated with smoking prevalence. Data from other populations have mixed results. In a cross-sectional study in Minnesota (91 % white; 52 % female), both aggregate level cohesion and individual social cohesion were associated with a lower likelihood of smoking.^26^ In a study among Asian-Americans in a California-based study (56 % female), men who had higher levels of neighborhood cohesion were less likely to smoke; however, there was no association among neighborhood cohesion and women.^8^ In a multiethnic sample (28 % African-American; 52 % female) from six US regions, individuals living in high socially cohesive neighborhoods were less likely to smoke.^22^

In our study, women with higher cohesion were older and had lived in the neighborhood longer. Higher social cohesion may be a marker of social inclusion and higher social status in the neighborhood.^39^ In subsidized housing, younger women likely have different social networks within the community than their older counterparts and are typically more transient. Women who have lived in neighborhoods for shorter time periods may have been more isolated, had less trust among neighbors, and/or experienced a different degree of embeddedness from their extended residential stability. Previous data have shown that residential instability in public housing disrupts social networks and supports^40^ which may impact social cohesion.

Stress has consistently been linked to smoking behaviors among women, as well as individuals living in public housing.^6^,^7^,^41^,^42^ Both individual perceptions of neighborhood stress (e.g., from violence and disorder) and living in higher stressed neighborhoods were associated with smoking in our study, which support findings from other studies among other diverse populations.^10^,^22^,^25^ Consistent with the social cohesion findings in this study, younger women also perceived higher stress from violence and disorder than their older counterparts. Younger women may have an increased exposure to neighborhood violence and disorder, as well as different social networks that expose them differently than older women. Younger women are also at higher risk for sexual assault and violence in public housing neighborhoods.^43^ Intimate partner violence is reported to be a significant problem in subsidized housing in qualitative studies,^24^,^44^ yet few studies have systematically examined women’s concerns in public housing about their experiences with crime victimization.^24^

This study was unique in that crime data were collected from the local police departments for the actual neighborhood, versus larger area census track data. Although perceived neighborhood disorder and exposure to violence were associated with smoking, actual crime proportions in the neighborhoods were not associated with smoking. Other studies assessing actual crime data and smoking prevalence in neighborhoods have mixed results. A study in the Southeastern USA found no association with neighborhood crime and smoking,^10^ while other studies outside the USA found positive associations.^45^,^46^ Further, the perceptions of crime and violence were not correlated with actual crime data in this study. It is important to note that the crime data were reported and analyzed for the year prior to the surveys in each respective neighborhood, while women in this study, on average, lived in their respective neighborhood for 5 years. Within each neighborhood, there were likely variances in police presence in the neighborhood, residents’ reporting of crime due to fear and retaliation, crime in the surrounding geographical community, and other historical events that may have contributed to the conduct and reporting of crimes.

Our study demonstrates that subsidized neighborhoods in a geographical region vary significantly from neighborhood to neighborhood with overall smoking prevalence, actual and perceived crime, disorder, social cohesion, number of residents, and length of residency (as shown in Table 2). These variances need consideration when designing randomized cluster trials and ecological based tobacco control interventions. Neighborhood social contextual factors not only affect tobacco consumption but also may affect the community’s ability to accept, mobilize, and promote adoption of health promoting interventions. Targeted and tailored tobacco control interventions are needed that consider both neighborhood level factors and individual level factors. Individual-based interventions are typically targeted to characteristics of the sample (e.g., sex and tobacco use) and then tailored to the individual characteristics of participants (e.g., behavioral cognitive skills training).^47^ Tailoring intervention strategies at the neighborhood level that address neighborhood characteristics (e.g., smoking prevalence, violence, disorder, and cohesion) with the goal of enhancing the adoption of health promoting behaviors are less understood. Individual and neighborhood strategies that may enhance social cohesion and mitigate neighborhood stress and the effect on health behaviors are not known in these high-risk neighborhoods and need further exploration. Tailored interventions and policies are needed to promote anti-smoking norms and disrupt the contagion effect of smoking among further generations.

### Limitations

Our study had several limitations. The study relied on self-reported data and the same source reporting bias for individual and neighborhood variables (with the exception of objective crime data) may have affected the significance of our results. The cross-sectional data limited our ability to make inferences about the neighborhood environment and smoking. Data on advertisements and availability of tobacco, which could potentially affect smoking prevalence in these neighborhoods, were not collected. This study was based in subsidized housing developments in the Southeastern USA and may not necessarily be generalized to other African-Americans in other regions. In addition, due to the number of neighborhoods (17) included in this study, inferences drawn from analyses including neighborhood level data were limited.

## Conclusions

This is the first known study to examine both individual and neighborhood level correlates among African-American women in subsidized housing developments. Our results not only add to a growing literature that neighborhood and social context influences health behavior but also further extends findings about associations between smoking behaviors and the physical and social environment in which individuals live. Future research should seek to explore differences among age groups and residential stability among these neighborhood level variables. Targeted and tailored multilevel, ecologically based interventions among this high-risk population are needed that address these complex individual and neighborhood contextual factors that may affect the individual’s and community’s success with tobacco control initiatives. Finally, policy implementation is needed to improve social determinants of health, especially poverty, neighborhood safety, and anti-smoking norms.
